# Comparing the Effects of Two Culture Methods to Determine the Total Heterotrophic Bacterial Colony Count in Hospital Purified Water

**DOI:** 10.1007/s44197-023-00186-1

**Published:** 2024-02-15

**Authors:** Xiongjing Cao, Huangguo Xiong, Yunzhou Fan, Lijuan Xiong

**Affiliations:** Department of Hospital Infection Management, Union Hospital, Tongji Medical College, Huazhong University of Science and Technology, 1277 Jiefang Ave, Wuhan, 430022 China

**Keywords:** Hospital purified water, Culture methods, Heterotrophic bacteria, Total bacterial number

## Abstract

**Background:**

Accurately detecting the quantity of microorganisms in hospital purified water is of significant importance for early identification of microbial contamination and reducing the occurrence of water-borne hospital infections. The choice of detection method is a prerequisite for ensuring accurate results. Traditional Plate Count Agar (PCA) belongs to a high-nutrient medium, and there may be limitations in terms of accuracy or sensitivity in detecting microorganisms in hospital purified water. On the other hand, Reasoner’s 2A agar (R2A) has characteristics, such as low-nutrient levels, low cultivation temperature, and extended incubation time, providing advantages in promoting the growth of aquatic microorganisms. This study, through comparing the differences in total colony counts between two detection methods, aims to select the method more suitable for the growth of aquatic microorganisms, offering new practical insights for accurately detecting the total count of heterotrophic bacteria in hospital purified water.

**Methods:**

The most commonly used plate count agar (PCA) method, and the R2A agar culture were adopted to detect microorganisms and determine the total number of bacterial colonies in the water for oral diagnosis and treatment water and terminal rinse water for endoscopes in medical institutions. The two water samples were inoculated by pour plate and membrane filtration methods, respectively. Using statistical methods including Spearman and Pearson correlation, Wilcoxon signed-rank sum test, paired-Chi-square test, and linear regression, we analyze the differences and associations in the bacterial counts cultivated through two different methods.

**Results:**

In 142 specimens of the water, the median and interquartile range of the heterotrophic bacterial colony number under the R2A culture method and under the PCA culture method were 200 (Q1–Q3: 25–18,000) and 6 (Q1–Q3: 0–3700). The total number of heterotrophic bacteria colonies cultured in R2A medium for 7 days was more than that cultured in PCA medium for 2 days (*P* < 0.05). The linear regression results showed a relatively strong linear correlation between the number of colonies cultured by the R2A method and that cultured by the PCA method (*R*^2^ = 0.7264). The number of bacterial species detected on R2A agar medium is greater than that on PCA agar medium.

**Conclusion:**

The R2A culture method can better reflect the actual number of heterotrophic bacterial colonies in hospital purified water. After logarithmic transformation, the number of colonies cultured by the two methods showed a linear correlation.

**Supplementary Information:**

The online version contains supplementary material available at 10.1007/s44197-023-00186-1.

## Introduction

Hospital purified water is widely utilized in clinical medical activities, as well as for the cleaning, disinfection, and sterilization of medical equipment and supplies. Contaminated hospital purified water can pose a risk of nosocomial cross-infections within healthcare facilities [1–4]. At present, the serious microbial contamination of hospital purified water has become a difficult problem faced by hospitals [5]. The contamination of hospital purified water can increase the risk of iatrogenic infection and the occurrence of related diseases, thus threatening the health of patients and medical staff [6, 7], especially those with basic diseases and low immunity, and even leading to death in severe cases [8, 9]. Research indicates that in a newly opened Neonatal Intensive Care Unit (NICU), there were two outbreaks of Pseudomonas aeruginosa infections over a span of more than 2 years. These outbreaks resulted in a total of 31 patient infections, with 3 fatalities. The primary cause of the outbreaks was contamination of the water supply pipes [10]. Some patients have died from infection with *Legionella* in the waterway during dental visits; a female patient succumbed to Legionnaires’ disease after receiving dental treatment. Upon investigation, *Legionella* were detected in the water discharged from the implicated high-speed turbine, and it was confirmed that the *Legionella* DNA genome found in the water was a complete match with the *Legionella* strain causing the patient’s infection [11]. Among the top ten medical technology hazards published by the Emergency Care Research Institute (ECRI) in 2016, gastrointestinal endoscopy is one of the medical devices most likely to cause nosocomial cross infection; however, the contamination of the terminal rinse water easily causes endoscope contamination, thus leading to iatrogenic infection [12, 13]. Therefore, it is of great significance to strengthen the microbiological monitoring of hospital purified water and attach importance to the strategy for detecting microorganisms in water for guiding the disinfection and quality control of hospital purified water and timely and effectively finding the source of infection and hidden danger of infection.

So far, the relevant research reports on microorganisms in hospital purified water have been mainly inclined to compare the total number of microbial colonies. However, there are relatively great differences in the detection methods for the total number of microbial colonies and also comparatively little relevant research. Currently, there is yet no uniform international standard for microbiological monitoring methods for hospital purified water [14]. Plate count agar (PCA) is the most commonly used medium for detecting the heterotrophic bacteria number in water in Heterotrophic Plate Counts (HPC). Comprising beef extract, peptone, sodium chloride, agar, and distilled water, PCA which is characterized by high-nutrient content has been used for the longest time in China. Also, it is the most widely applied medium. However, some studies have shown that the evaluation of microbial indexes in water and the cleaning and disinfection effect of waterways and pipe networks based on PCA counting results may not be complete, with the degree of microbial contamination in water possibly beyond people’s expectations [3, 6].

Research indicates that using R2A agar medium to detect the quantity of microorganisms in water yields good results [15–18]. However, it still needs further study whether there is a correlation between the total heterotrophic bacteria number detected by R2A and PCA media. In terms of composition, R2A agar medium includes glucose, potassium dihydrogen phosphate, sodium pyruvate, magnesium sulfate, soluble starch, peptone, beef extract, yeast extract, agar, distilled water, and more. Compared to PCA agar medium, R2A agar medium has significantly reduced nutrient content, but a broader range of carbon sources. As a low-nutrient medium, it may be more suitable for the growth of heterotrophic bacteria in nutrient-poor aquatic environments. However, more practical evidence is needed. Regarding cultivation conditions, PCA agar medium is cultured at 36 °C ± 1 °C for 48 h. This cultivation temperature is designed based on the “body temperature” and may not be suitable for all aerobic and facultative anaerobic heterotrophic bacteria in aquatic environments. The R2A agar medium is employed for the enumeration of heterotrophic bacteria in water, and the incubation is carried out at a temperature range of 17 °C to 23 °C for a period of 7 days [19], Low-temperature, extended incubation favors bacterial growth in aquatic environments [20].

Current research predominantly relies on the conventional plate counting techniques for microbial inoculation. In addition to employing the conventional plate count method, our study also utilized the internationally advanced membrane filtration method. By comparing the differences in heterotrophic bacterial counts detected using R2A and PCA culture media under different testing methods, we aimed to enhance the accuracy and generalizability of the results. The approach contributes valuable insights for healthcare institutions seeking more precise microbial analysis in hospital purified water, offering reference and guidance for advanced water quality assessment methods.

## Methods

### Research Design and Objects

The water for three-purpose guns in the stomatological comprehensive treatment table in the Department of Stomatology of a large tertiary A-level hospital and for terminal rinse for endoscopes in the endoscopy center was selected as the research objects by random sampling methods. Pour plate and membrane filtration were adopted to detect the water for the oral cavity and the terminal rinse water for endoscopes, respectively. PCA and R2A media were used for their culture according to the corresponding culture conditions.

### Data Collection

During the period from January to May 2022, a total of 192 specimens were collected, among which 79 were the water for the three-purpose gun of the oral comprehensive treatment table and 113 were the terminal rinse water for endoscopes. The sampling methods were as follows. According to the aseptic operation principles, sterile containers were used for direct sampling. The sterile three-purpose gun head was replaced at the time of sampling the water for the oral three-purpose gun, followed by pressing the water-spraying button of the three-purpose gun for discharging water continuously for 30 s, and the sterile sampling tube was used to collect 10 ml discharge water from the three-purpose gun for detection. In sampling the terminal rinse pure water for endoscopes, the water was discharged continuously for 30 s, followed by the collection of 200 ml in a sterile sampling bottle for detection. After sampling, the specimens were stored in a low-temperature refrigerator and sent for detection, and all samples were detected within 2 h after collection.

### Sample Analysis

All petri dishes used in this study were commercially available and obtained from Chongqing Pangtong Company. They were used within their expiration date, and corresponding acceptance and quality control procedures were strictly followed. Attention was given to the moisture content on the agar surface, and if cracks appeared, even within the expiration period, the dishes were not used. The PCA agar medium is composed of beef extract, peptone, sodium chloride, agar, and distilled water. The R2A agar medium consists of glucose, potassium dihydrogen phosphate, sodium pyruvate, magnesium sulfate, soluble starch, peptone, beef extract, yeast extract, agar, and distilled water.

Detection methods were as follows. In detecting the water for oral diagnosis and treatment, 1.0 ml each of tenfold and 100-fold dilutions of the stock solution of the water sample was taken on the ultra-clean workbench, followed by pouring PCA and R2A agar into them, respectively, with parallel samples set for each concentration. In addition to following the above method for inoculating the terminal rinse water for endoscopes, 20 and 100 ml of it were taken for membrane filtration with 0.45 μm film, respectively, with the filter membrane stuck on the agar medium. A blank control was set for each medium. The culture temperature and duration were in accordance with the international recommended standards. The PCA medium was placed in a 36 °C ± 1 °C incubator for 48 h culture. And the R2A medium was placed in a 17 °C–23 °C incubator for 168 h (7 d) culture for colony counting. In our investigation, specimens underwent mass spectrometry analysis using the fully automated rapid microbial identification system (VITEK MS).

### Results’ Evaluation

Results were judged as follows. The bacteria counting of the water for oral diagnosis and treatment were mainly based on the number of colonies actually growing out on the culture medium. After the terminal rinse water of endoscopes was cultured, if the filter membrane method failed to be used for counting, the results detected by the pour plate method should prevail; if the filter membrane method could be used for counting, the sum of the number of colonies on both the plate and filter membrane was counted. The colony number of the terminal rinse water for endoscopes was judged according to China’s “Technical Specifications for Cleaning and Disinfection of Flexible Endoscopes (WS507-2016)” [21], and the total bacterial colony number ≤ 10 CFU/100 ml was judged qualified. The qualified rate of the water for oral diagnosis and treatment was calculated according to the standards ≤ 500 CFU/m and ≤ 100 CFU/ml, respectively [22–24].

### Data Analysis

The mean ± standard deviation, median, and interquartile range were used to describe the distribution situation of heterotrophic bacterial colonies growing on PCA and R2A media of water sample specimens from the oral cavity and the endoscope (after the original value and logarithm transformation); Shapiro test was adopted to test the normality of data; Spearman and Pearson correlation was adopted to analyze the correlation between the number of colonies growing under the PCA and R2A culture methods were performed. Wilcoxon signed-rank sum test was used to compare the number of colonies under the two culture methods between groups; the qualified rate of the culture results under the two culture methods was compared by paired-chi square test. Linear regression was adopted to investigate the correlation between the number of colonies (after logarithm transformation) cultured by two culture methods. All statistical analyses were completed by SAS version 9.4.

## Results

### Bacteria Counting Under the Two Culture Methods

Among the 192 water specimens, no colony was detected in 50 by PCA and R2A culture methods, and the data on these 50 specimens were excluded from the analyses of colony number difference and linear correlation. Among the 50 samples without bacterial growth, 40 were from endoscope terminal rinsing water, and 10 were from oral dental floss water. Among the remaining 142 water specimens, 53 were oral specimens and 89 were endoscopic ones. In PCA agar samples, detection revealed the presence of *Corynebacterium*, *Burkholderia contaminants*, and *Burkholderia gladioli*. Meanwhile, R2A agar samples exhibited the presence of *Burkholderia contaminants*, *Brevundimonas vesicularis*, *Ralstonia pickettii*, *Sphingomonas paucimobilis, Gluconacetobacter*, and *Herbaspirillum huttiense*.

The mean ± standard deviation of the colony number of 142 specimens under the PCA culture method was 8949.18 ± 23,375.39, with the median and interquartile range of 6 (Q1–Q3: 0–3700); the mean ± standard deviation of the colony number of specimens under the R2A culture method was 24,733.00 ± 59,407, with the median and interquartile range of 200 (Q1–Q3: 25–18,000) (Table 1). Among them, the mean ± standard deviation of the colony number of the water for oral diagnosis and treatment under the PCA culture method was 13.09 ± 28.72, with the median and interquartile range of 0 (Q1–Q3: 0–14); the mean ± standard deviation of the colony number of the water for oral diagnosis and treatment under the R2A culture method was 260.38 ± 553.19, with the median and interquartile range of 70 (Q1–Q3: 20–210) (Supplementary information Table 1); the mean ± standard deviation of the colony number of the terminal rinse water for endoscopes under the PCA culture method was 14,270.66 ± 28,262, with the median and interquartile range of 1500 (Q1–Q3: 0–16,900); the mean ± standard deviation of the colony number of the terminal rinse water for endoscopes under the R2A culture method was 39,306.58 ± 71,267, with the median and interquartile range of 9000 (Q1–Q3: 32–35,000) (Supplementary information Table 2); the number of colonies cultured by both two culture methods did not meet the normal distribution (*P* < 0.05), and further statistical analysis was made after logarithm transformation of the data.

**Table 1:** Statistical description of the number of colonies in PCA and R2A in all samples (*N* = 142)

Variable	R2A day7	PCA day2	Log (R2A day7)	Log (PCA day2)
Mean	24,733	8949.18	6.15	3.85
SD	59,407	23,375.39	3.68	4.3
Median	200	6	5.3	1.79
Q1–Q3	25–18,000	0–3700	3.21–9.80	0–8.22
Shapiro test *P *value	< 0.0001	< 0.0001	< 0.0001	0.0002
Wilcoxon signed-rank test *P *value	< 0.0001		< 0.0001	

### Differential Analysis of the Colony Number Between R2A and PCA Culture Methods

After the logarithm transformation of data, the median and interquartile range of the colony number of 142 water samples under the PCA culture method was 3.85 (Q1–Q3: 0–8.22), and the median and interquartile range of the colony number under the R2A culture method was 6.15 (Q1–Q3: 3.21–9.80). Wilcoxon signed-rank sum test showed that the number of colonies cultured in the R2A medium for 7 days was higher compared with the PCA medium for 2 days, with a statistically significant difference(*P* < 0.05) (Table 1). When the water for oral diagnosis and treatment and the terminal rinse water for endoscopes were separated for comparison, the results all showed that the number of colonies cultured under the R2A culture method was higher compared with the PCA culture method, with a statistically significant difference (Supplementary information Tables 1 and 2).

### The Linear Correlation Between the Number of Colonies Growing Under the R2A Culture Method and That of Colonies Growing in the PCA Culture Method

After logarithm transformation, the Spearman correlation coefficient of the number of colonies cultured under two culture methods in 142 water samples was 0.85 (95% CI 0.80–0.89), with the Pearson correlation coefficient of 0.90 (95% CI 0.86–0.93). There was a strong correlation between the two culture results under R2A and PCA culture methods. When the terminal rinse water for endoscopes was separated from the water for oral diagnosis and treatment for the correlation analysis of the colony number under two culture methods, the analysis of the culture results of the terminal rinse water for endoscopes showed that there was still a strong correlation between the two, with the Spearman correlation coefficient of 0.85 (95% CI 0.77–0.90) and the Pearson correlation coefficient of 0.92 (95% CI 0.88–0.95); the analysis of the culture results of the water for the oral diagnosis and treatment showed a relatively weak correlation between the two, with the Spearman correlation coefficient of 0.45 (95% CI 0.20–0.64) and Pearson correlation coefficient of 0.47 (95% CI 0.22–0.65) (Table 2).

**Table 2:** Correlation coefficient of the number of colonies between PCA and R2A culture dishes

Correlation coeffs with 95% confidence bounds						
Coefficient correlation	All sample (*N* = 142)		Oral cavity water (*N* = 53)		Endoscopic water sample (*N* = 89)	
Spearman	0.85	0.80–0.89	0.45	0.20–0.64	0.85	0.77–0.90
Pearson	0.90	0.86–0.93	0.47	0.22–0.65	0.92	0.88–0.95

**Fig. 1:**
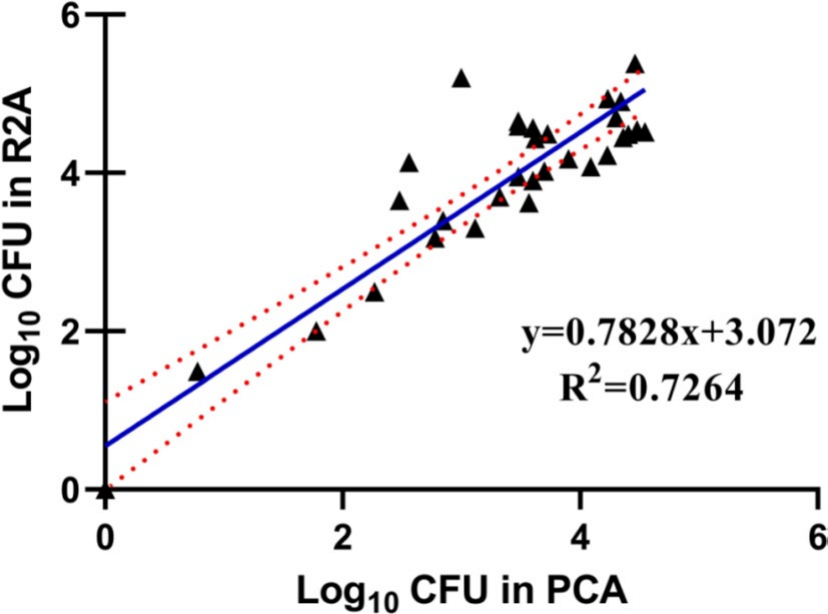
Scatter plots and linear regression results of PCA and R2A Culture dish cultures for all samples

The linear regression results showed that after logarithm transformation, there was in general a relatively strong linear correlation between the number of colonies cultured by R2A and PCA culture methods (Fig. 1, *R*^2^ = 0.7264). The analysis of the culture results of the water for oral diagnosis and treatment showed a relatively weak linear correlation between the two (supplementary information Fig. 1, *R*^2^  =  0.2576). However, the analysis of the culture results of the terminal rinse water for endoscopes still showed a relatively strong linear correlation between the two (supplementary information Fig. 2, *R*^2^ = 0.7775).

### Under Different Standards, the Unqualified Rates Detected Under the R2A Culture Method All Higher Compared with the PCA Culture Method

The paired-Chi-square test was performed on the colony number growth values of the water for oral diagnosis and treatment and the terminal rinse water for endoscopes under two culture methods, respectively. When the qualified critical value of the water for the oral diagnosis and treatment was ≤ 100 CFU/mL or ≤ 500 CFU/mL, the unqualified rates of detected results under the R2A culture method were all higher compared with the PCA culture method, with a statistically significant difference (*P* < 0.05) (Table 3 and supplementary information Table 3); similarly, the unqualified rate of the detected results of the terminal rinse water for endoscopes under the R2A culture method was also higher compared with the PCA culture method, with a statistically significant difference (*P* < 0.05) (Table 4).

**Table 3:** Paired Chi-square test for the qualified rate of oral water culture in PCA and R2A culture dishes (with 100 as the critical value)

R2A day7（CFU/mL） PCA day2 （CFU/mL)	Eligible (≤ 100)	Exceed standard (≥ 100)	Summary	*P* value
Eligible (≤ 100)	59	19	78	< 0.0001
Exceed standard (> 100)	0	1	1	
Summary	59	20	79

**Table 4:** Paired-Chi-square test for the qualified rate of endoscopy hydroculture in PCA and R2A culture dishes

R2A day7 (CFU/100 mL) PCA day2 (CFU/100 mL)	Eligible (≤ 10)	Exceed standard (> 10)	Summary	*P* value
Eligible (≤ 10)	41	16	57	< 0.0001
Exceed standard (> 10)	0	56	56	
Summary	41	72	113	

## Discussion

Increasingly, hospital purified water has been identified as the cause of biological risk in the hospital practice. Some studies show that water is the vehicle through which most of the infections that develop in the dental office are spread [9, 25]. Regular microbial testing of hospital purified water is an essential measure to prevent bacterial colonization and biofilm formation, thereby avoiding the occurrence of water-borne infections [26, 27]. This study showed that the use of the PCA medium for the detection of microbial contamination in water might have an underestimation of the real number of heterotrophic bacteria, thus causing false negatives. The water for oral diagnosis and treatment and the terminal rinse water for endoscopes were inoculated by pour plate and membrane filtration methods, respectively. The number of heterotrophic bacteria in water samples could be detected after both 2 days of culture with R2A medium and 7 days of culture with PCA medium. However, no matter what kind of water samples and which inoculation method was used, the unqualified rates detected under the R2A culture method were all significantly higher compared with the PCA culture method. The total number of heterotrophic bacteria detected under the R2A culture method was significantly higher compared with the PCA culture method. It could be seen that the sensitivity of the R2A culture method for the detection of microorganisms in water is higher.

The CDC of the United States and the Australian Dental Association recommend that the total aerobic heterotrophic bacterial colony number of the water for oral diagnosis and treatment should be ≤ 500 CFU/m [22]. And it is ≤ 100 CFU/mL in the EU countries [23], and the Chinese reference hygienic standard for drinking water hygiene standard is ≤ 100 CFU/ml [24]. The qualified rate of the water for oral diagnosis and treatment was compared by us with reference to different standards. And it was found that under different limit values, the unqualified rates of the total heterotrophic bacterial colony number detected by the PCA culture method were all lower compared with the R2A culture method. The standard of ≤ 10 CFU/100 ml was referred for the terminal rinse water for endoscopes [21]. And the unqualified rates of the total heterotrophic bacterial colony number detected by the PCA culture method were all lower compared with the R2A culture method. It was suggested that the PCA medium may not be sensitive enough for the detection of heterotrophic bacteria in hospital purified water. This may be related to the following reasons: PCA medium, a high-nutrient medium, is relatively suitable for heterotrophic bacteria with high nutritional requirements and rapid growth. However, this category of bacteria accounts for a relatively small part of bacterial species in the water sample, whereas a relatively large part of oligotrophic heterotrophic bacteria are not adapted to the high-nutrient medium, and fail to grow and reproduce within the set time to form colonies visible to the naked eye, the high-nutrient medium may also inhibit or place metabolic stress on nutrient-limited water populations [27].

From the research results, the R2A culture method is more suitable for the detection of microorganisms in hospital purified water. R2A medium was recommended for the detection of aquatic microorganisms in some studies [9, 28], whereas there are few studies of the R2A medium for the detection of heterotrophic bacteria in hospital purified water. However, the waterway system of hospital purified water is more complex, and easier to be contaminated. Moreover, the disinfection treatment measures are also different from tap water. This study showed that the detection sensitivity of the R2A culture method was all better for the water for oral diagnosis and treatment and the terminal rinse water of endoscopes in medical institutions, compared with the PCA culture method. The reasons were as follows: the R2A culture medium belongs to a low-nutrition culture medium, with a relatively wide range of nutritional composition; in addition, setting a relatively low culture temperature and an extended culture time is more suitable for the activation and culture of inhibited bacteria in water samples [29]. In contrast to bacteria on PCA agar, which can form visible colonies in a short timeframe, bacteria on R2A agar exhibit slower growth rates, resulting in smaller colonies. Therefore, an extended incubation period is required to allow the colonies to grow sufficiently large for ease of counting. Moreover, chemical disinfectants are used for regularly disinfecting the waterways and pipeline systems to curb the growth of biofilm in medical institutions [30]. From the R2A formula, sodium pyruvate in the R2A culture medium, an antioxidant, can promote restorative regrowth of damaged bacteria damaged by disinfectants. The soluble starch in R2A can absorb harmful substances produced during the resuscitation process of heterotrophic bacteria [31]; hence, the total count of heterotrophic bacteria on R2A agar is consistently higher than that on PCA agar. And it is more suitable for the detection of hospital purified water.

At present, there is yet no uniform international standard for the detection of hospital purified water, and the detection methods used by countries and even regions are inconsistent [20]. This results in the failure to compare the detection results between regions, and compare the microbial status quo of hospital purified water horizontally. However, after logarithm transformation of the number of heterotrophic bacteria detected in the two media, there was a high linear correlation between the number of colonies cultured in the R2A and PCA media, which was consistent with the research results of Bugno, A, etc. [32]. We can infer their growth situation on the R2A medium from the total number of heterotrophic bacteria growing on the PCA medium. The specific calculation method needs to be further simulated and calculated by increasing the sample size.

Research indicates that the majority of literature on water-borne pathogens originates from Europe or the United States, with limited reports from developing countries [8]. The findings of our study contribute to understanding the bacterial species in hospital purified water in developing countries. In this investigation, the diversity of heterotrophic bacteria detected on R2A agar exceeded that on PCA agar. While *Corynebacterium* and *Burkholderiaceae* were identified in PCA agar samples, heterotrophic bacteria on R2A agar were primarily classified into *Pseudomonadaceae*, *Burkholderiaceae*, *Ralstonia spp*, *Acetobacteraceae*, and *Herbaspirillum*. This aligns with common bacteria isolated and identified in DUWLs, as reported in the other study [33].

The presence of heterotrophic bacteria in hospital water poses certain biosecurity risks, threatening patient health and leading to infections. *Burkholderia* contamination in dentures may potentially result in pneumonia [34]. *Burkholderia gladioli*, a non-fermenting Gram-negative rod-shaped aerobe, has been reported in the literature primarily in immunocompromised adults and neonates in cases of gladiolus infection [35]. *Ralstonia pickettii* is not considered a major pathogen, exhibiting relatively low virulence. However, infections caused by this bacterium have been reported, including instances of bloodstream infection [36], meningitis [37], and contamination in Plasmodium in vitro cultures [38]. *Brevundimonas vesicularis*, a Gram-negative rod, rarely causes human infections, but there are studies reporting cases of chronic peritoneal dialysis patients developing Sphingomonas paucimobilis peritonitis [39]. As a nitrogen-fixing bacterium, H*erbaspirillum huttiense* has been implicated in the first reported case of sepsis in Korea caused by this organism [40]. *Gluconacetobacter* and *Sphingomonas paucimobilis* have limited literature reporting human infections. Future investigations can employ more detailed analyses, such as colony counts and strain typing, to gain deeper insights into the microbial diversity and abundance in hospital purified water.

The R2A culture method had higher sensitivity for the detection of microorganisms in hospital purified water. This will provide reference and guidance for medical institutions to carry out more accurate microbial detection on hospital purified water, with great significance for the timely and effective detection of hidden dangers of water-borne infection. There were the following limitations in this study. First, the acquisition time of oral samples was all within 7 days after the pipes of the oral comprehensive treatment table were disinfected. Therefore, the total heterotrophic bacterial colony number was relatively low compared with other studies. Second, this study, which was carried out in the provincial capital of Hubei Province, China, was not verified in other cities. Therefore, it was not ruled out that the detection rates under the R2A culture method could be all affected due to regional differences, the concentration of organic substances in water bodies, and the characteristics of the category, physiology, biochemistry, etc. of the primary contaminated bacteria. The research needs to be confirmed in more regional studies in the future.

## Conclusion

Compared to the PCA culture method, the R2A culture method exhibits higher sensitivity, providing a more accurate reflection of the actual colony counts of heterotrophic bacteria in hospital purified water. It is recommended to conduct parallel specimens using the R2A method for key water departments in medical institutions (such as dental departments, endoscopy centers, disinfection supply centers, operating rooms, etc.). In circumstances where feasible, parallel testing with the PCA method should be carried out simultaneously, with a more detailed examination of colony counts, strain typing, and other aspects. This comprehensive approach aims to thoroughly understand the contamination of heterotrophic bacteria in medical institution water sources, gradually establishing an evaluation system and quality control standards for the R2A detection method.

The selected subjects in this study are highly representative, and the comparison of detection results under different inoculation methods enhances the generalizability of the research findings. Additionally, the study identifies a linear correlation between the total colony counts of heterotrophic bacteria on PCA and R2A agar, providing a basis for comparing data from different detection methods across regions. However, due to the diversity of physiological and biochemical characteristics of bacteria, it is necessary to expand the scope of future research and further investigate the applicable scenarios and target bacteria for the R2A culture method.

### Supplementary Information

Below is the link to the electronic supplementary material.

Supplementary file1 (PDF 109 KB)

Supplementary file2 (PDF 111 KB)

Supplementary file3 (PDF 121 KB)

Supplementary file4 (PDF 133 KB)

Supplementary file5 (PDF 132 KB)

## Data Availability

The datasets during and/or analyzed during the current study are available from the corresponding author on reasonable request.

## Abbreviations

PCA: Plate count agar
ECRI: Emergency care research institute
HPC: Heterotrophic plate counts
