# Supplementary material for: Comparing the Effects of Two Culture Methods to Determine the Total Heterotrophic Bacterial Colony Count in Hospital Purified Water
Source: J Epidemiol Glob Health. 2024 Feb 15;14(1):184–92. doi: 10.1007/s44197-023-00186-1 (PMC11043230; doi:10.1007/s44197-023-00186-1)
Supplement: Supplementary file 1 — Supplementary file1 (PDF 109 KB) [file 44197_2023_186_MOESM1_ESM.pdf]

Scatter plot and linear regression of PCA and R2A culture cultures in oral samples

Title: Comparison of Effects of Two Culture Methods for Determining the Total Heterotrophic Bacterial Colony Number in Medical Water

Journal : Current Environmental Health Reports

Authors: Cao Xiongjing [a*], Xiong Huangguo [a*], Fan Yunzhou [a*], Xiong Lijuan [a]

Affiliation: Department of Hospital Infection Management, Union Hospital Affiliated to Tongji Medical College of Huazhong University of Science and Technology , Wuhan.

Email: lijuanxiong2016@126.com

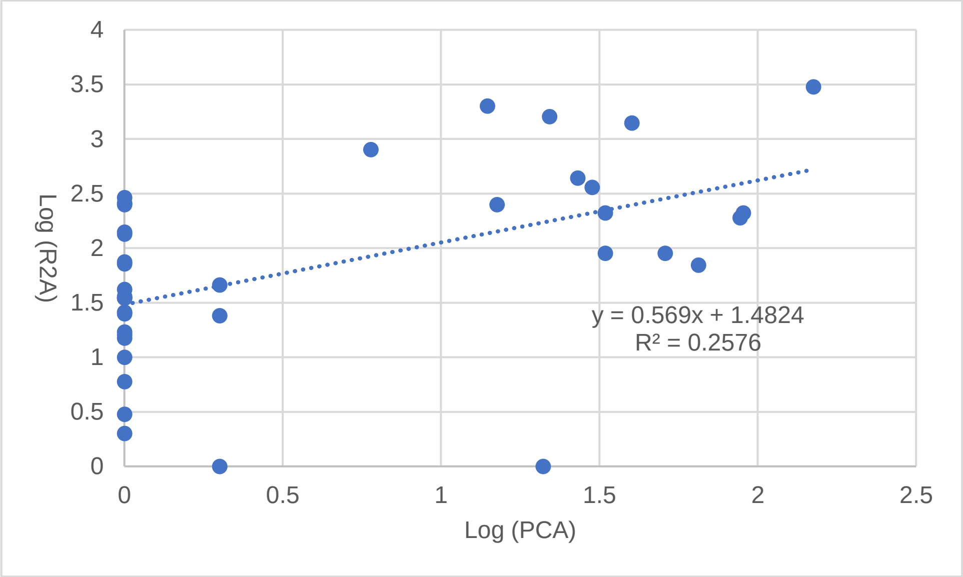

Fig.1 Scatter plot and linear regression of PCA and R2A culture cultures in oral samples
