# Supplementary material for: Comparing the Effects of Two Culture Methods to Determine the Total Heterotrophic Bacterial Colony Count in Hospital Purified Water
Source: J Epidemiol Glob Health. 2024 Feb 15;14(1):184–92. doi: 10.1007/s44197-023-00186-1 (PMC11043230; doi:10.1007/s44197-023-00186-1)
Supplement: Supplementary file 3 — Supplementary file3 (PDF 121 KB) [file 44197_2023_186_MOESM3_ESM.pdf]

Statistical description of the number of colonies in PCA and R2A in oral samples

Title: Comparison of Effects of Two Culture Methods for Determining the Total Heterotrophic Bacterial Colony Number in Medical Water

Journal : Current Environmental Health Reports

Authors: Cao Xiongjing[a*], Xiong Huangguo[a*], Fan Yunzhou[a*], Xiong Lijuan[a]

Affiliation: Department of Hospital Infection Management, Union Hospital Affiliated to Tongji Medical College of Huazhong University of Science and Technology, Wuhan.

Email: lijuanxiong2016@126.com

Table1 Statistical description of the number of colonies in PCA and R2A in oral samples **(N=53)**

Variable	R2A day7	PCA day2	Log (R2A day7)	Log (PCA day2)
**mean**	260.38	13.09	4.05	1.03
**SD**	553.19	28.72	1.92	1.64
**median**	70.00	0	4.25	0
**Q1-Q3**	20-210	0-14	3.00-5.35	0-2.64
**Shapiro-test P-value**	<0.0001	<0.0001	0.5248	<0.0001
**Wilcoxon Signed Ranks Test P-value**	<.0001		<.0001	
