# Supplementary material for: Comparing the Effects of Two Culture Methods to Determine the Total Heterotrophic Bacterial Colony Count in Hospital Purified Water
Source: J Epidemiol Glob Health. 2024 Feb 15;14(1):184–92. doi: 10.1007/s44197-023-00186-1 (PMC11043230; doi:10.1007/s44197-023-00186-1)
Supplement: Supplementary file 4 — Supplementary file4 (PDF 133 KB) [file 44197_2023_186_MOESM4_ESM.pdf]

Statistical description of the number of colonies in PCA and R2A in endoscopy samples

Title: Comparison of Effects of Two Culture Methods for Determining the Total Heterotrophic Bacterial Colony Number in Medical Water

Journal : Current Environmental Health Reports

Authors: Cao Xiongjing [a*], Xiong Huangguo [a*], Fan Yunzhou [a*], Xiong Lijuan [a]

Affiliation: Department of Hospital Infection Management, Union Hospital Affiliated to Tongji Medical College of Huazhong University of Science and Technology , Wuhan.

Email: lijuanxiong2016@126.com

Table2 Statistical description of the number of colonies in PCA and R2A in endoscopy samples **(N=89)**

Variable	R2A day7	PCA day2	Log (R2A day7)	Log (PCA day2)
**mean**	39306.58	14270.66	7.39	5.53
**SD**	71267	28262	3.92	4.52
**median**	9000	1500.00	9.10	7.31
**Q1-Q3**	32-35000	0-16900	3.47-10.46	0-9.74
**Shapiro-test P-value**	<.0001	<0.0001	<0.0001	<0.0001
**Wilcoxon Signed Ranks Test P-value**	<.0001		<.0001	
