# Supplementary material for: Comparing the Effects of Two Culture Methods to Determine the Total Heterotrophic Bacterial Colony Count in Hospital Purified Water
Source: J Epidemiol Glob Health. 2024 Feb 15;14(1):184–92. doi: 10.1007/s44197-023-00186-1 (PMC11043230; doi:10.1007/s44197-023-00186-1)
Supplement: Supplementary file 5 — Supplementary file5 (PDF 132 KB) [file 44197_2023_186_MOESM5_ESM.pdf]

Paired Chi-square test for the Qualified rate of oral water Culture in PCA and R2A Culture dishes

Title: Comparison of Effects of Two Culture Methods for Determining the Total Heterotrophic Bacterial Colony Number in Medical Water

Journal : Current Environmental Health Reports

Authors: Cao Xiongjing [a*], Xiong Huangguo [a*], Fan Yunzhou [a*], Xiong Lijuan [a]

Affiliation: Department of Hospital Infection Management, Union Hospital Affiliated to Tongji Medical College of Huazhong University of Science and Technology , Wuhan.

Email: lijuanxiong2016@126.com

Table3 Paired Chi-square test for the Qualified rate of oral water Culture in PCA and R2A Culture dishes (with 500 as the critical value)

**PCA day2**	**R2A day7**		**Summary**	**P-value**
	**Eligible (≤500)**	**Exceed standard (＞500)**		
**Eligible (≤500)**	73	5	78	
**Exceed standard (＞500)**	0	1	1	<0.0253
**合计**	73	6	79	
